# Whole-embryonic identification of maternal microchimeric cell types in mouse using single-cell RNA sequencing

**DOI:** 10.1038/s41598-022-20781-9

**Published:** 2022-11-04

**Authors:** Kana Fujimoto, Akira Nakajima, Shohei Hori, Yumiko Tanaka, Yoshitaka Shirasaki, Sotaro Uemura, Naoki Irie

**Affiliations:** 1 Department of Biological Sciences, Graduate School of Science, University of Tokyo, Hongo, Bunkyo-ku, Tokyo, 113-0033 Japan; 2 Graduate School of Pharmaceutical Sciences, University of Tokyo, Bunkyo-ku, Tokyo, Japan; 3 Universal Biology Institute, University of Tokyo, Bunkyo-ku, Tokyo, Japan

**Keywords:** Developmental biology, Immunology

## Abstract

Even though the mother and the fetus of placental mammals are immunologically non-self with respect to one other, mutual exchange of small numbers of cells between them is known to occur. Maternal cells entering the fetus, called maternal microchimeric cells (MMc cells), are thought to be involved in different physiological phenomena, such as establishing immune tolerance, tissue repair, and the pathogenesis or deterioration of some inflammatory diseases and congenital malformations. While specific MMc cell types have been reported as associated with these phenomena, the contribution of MMc cells to these different outcomes remains unknown. As one possibility, we hypothesized that different embryos have differing repertoires of MMc cell types, leading to or biasing embryos toward different fates. To date, no studies have succeeded in identifying the MMc cell type repertoire of a single embryo. Accordingly, here, we isolated MMc cells from whole mouse embryos, determined their types, and analyzed their MMc cell type variability. By combining our previously established, whole-embryonic MMc isolation method with single-cell RNA sequencing, we successfully estimated the cell type repertoires of MMc cells isolated from 26 mouse embryos. The majority of MMc cells were immune-related cells, such as myeloid cells and granulocytes. We also detected stem cell-like MMc cells expressing proliferation marker genes and terminally differentiated cells. As hypothesized, we noted statistically significant inter-individual variation in the proportion of immune-related cells in the different embryos. We here successfully estimated MMc cell types in individual whole mouse embryos. The proportion of immune-related cells significantly differed among the individual embryos, suggesting that the variations are one of the potential mechanisms underlying the differing MMc-related physiological phenomena in offspring. These findings provide insight into cell-level epigenetics by maternal cells.

## Introduction

Placental mammals, such as humans and mice, are chimeric by nature, with a small number of maternal cells in the body^1–3^. These maternal cells originate mainly from cells that had migrated into the fetus during pregnancy and persist for decades or, most likely, for the entirety of one’s life^4^. This renders individuals chimeric, or “microchimeric”, as these cells are genetically and immunologically non-self to the progenitor^1–3^. This phenomenon is called maternal microchimerism, with maternal cells called maternal microchimeric (MMc) cell. Considering the mother-to-fetus inheritance of maternal cells, microchimerism can be regarded as cell-level epigenetics.

While MMc cell migration to the fetus is thought to occur in all pregnancies, the potential biological roles of MMc cells concern phenomena related to health as well as disease. Increased frequency of MMc cells has been implicated in such physiological phenomena as the establishment of immune tolerance against non-inherited maternal antigens in offspring^5–8^, regeneration^4,9–11^, and the pathogenesis or deterioration of some inflammatory diseases and congenital malformations^12–16^. Based on observations of maternal cell enrichment in different phenomena, MMc cells are thought to potentially play both beneficial and harmful roles. For instance, MMc cells were identified as pancreatic insulin-producing β cells in an individual with type 1 diabetes, suggesting that they might help regenerate damaged β cells of a child, at least partially^9,11^. In contrast, enriched numbers of maternal cytotoxic T cells were reported in a damaged liver in an individual with biliary atresia^12,13^. As cytotoxic T cells can attack the immunologically non-self-antigens, and the frequency of MMc cells tends to be higher in individuals with biliary atresia than in control individuals with other diseases, MMc cells may cause or enhance the inflammatory aspect of this congenital disease^12,13^. However, of note, studies to date have only demonstrated a correlation between a disease and increased frequency of MMc cells, together with an increased immunological compatibility between the mother and the diseased individual^14^, with no direct evidence to indicate that MMc cells are the actual cause of certain congenital diseases.

Considering the available data, it would be tempting to speculate on the association of MMc cells with such various physiological phenomena as tolerance, regeneration, and possible tissue damage in the fetus. One possibility is that the MMc cell type repertoire is different in individual embryos, leading to or biasing the embryo toward different outcomes. In this regard_,_ while various MMc cell types have been reported, such as immune-related cells, intrahepatic biliary epithelial cells, insulin cells, and stem cells^3,4,17–19^, no studies to date have succeeded in dissecting the MMc cell type repertoire of single embryos to test the above possibility. In other words, we still do not know which types of cells constitute the major proportion of MMc cells that migrate in the fetal body.

We have previously reported developing a method for detecting live MMc cells in the individual whole mouse embryo^20^, by taking advantage of flow cytometric cell sorting and transgenic mice expressing green fluorescent protein (GFP^21^). Using this technique, we found that the number of MMc cells differs between individual fetuses, including an embryo at the latest developmental stage containing approximately 500 times more MMc cells than other embryos.

In the present study, we isolated MMc cells from whole mouse embryos and estimated their types to elucidate the MMc cell type repertoires and to test the hypothesis that the repertoires differ among individual embryos. In short, by combining the previously developed MMc cell isolation technique with single cell RNA-seq (scRNAseq), we estimated the repertoire of MMc cell types in the early phase (E14.5) of MMc cell migration. This was to minimize the bias from counting cell types that were differentiated from multipotent progenitor MMc cells. While definitive identification of cell types based on scRNAseq is difficult, it still allows for a more comprehensive estimation of cell types compared with previous approaches that utilizes a limited set of antibodies. Our results indicated that the majority of MMc cells were estimated to be the immune-related cell types, such as myeloid cells, granulocytes, and T cells. Furthermore, we also found a substantial proportion of other cell types including terminally differentiated and progenitor cells.

## Results

### Isolation of GFP-positive MMc cells from mouse embryos

**Figure 1:**
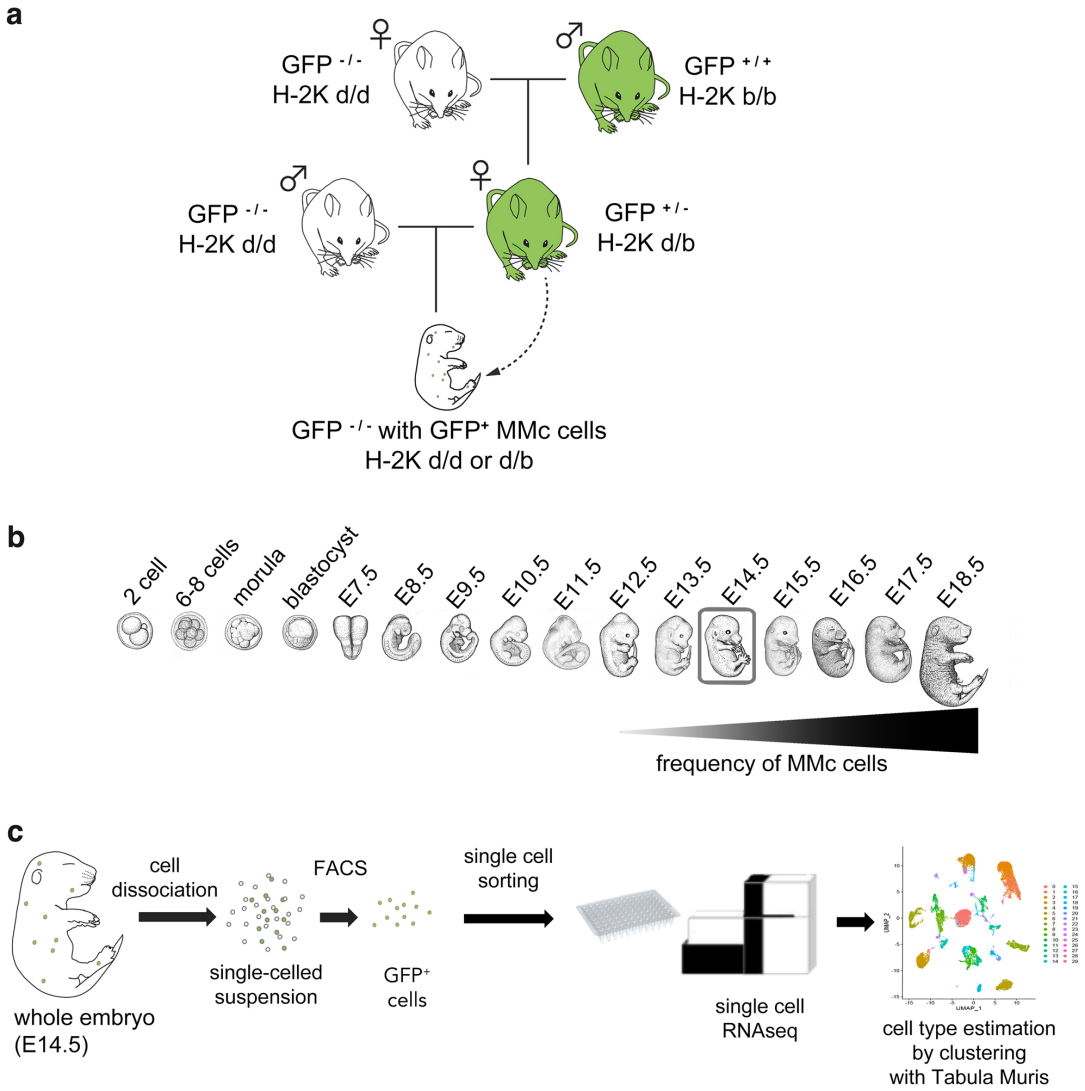
Experimental design for MMc cell isolation and cell type estimation. (**a**) To label MMc cells with GFP, transgenic mouse line [C57BL/6-Tg(CAG-EGFP)C14-Y01-FM131Osb] expressing GFP systemically was used. To avoid detecting grand-maternal cells as MMc cells^22^, *GFP*-heterozygous female mouse was first obtained by mating wild-type female mouse and a *GFP*-homozygous transgenic male mouse. Then, the *GFP*-heterozygous female, the mother mouse was mated with wild-type male mouse to obtain GFP-negative fetuses, which contained GFP-positive MMc cells. In addition, MMc cells were enriched based on differences in the major histocompatibility complex (MHC) using magnetic cell sorting: in the *GFP*-heterozygous mother mouse, H-2Kd/b was displayed as MHC class II molecule on the cell surface; in the fetus, H-2Kd/d or d/b would be displayed and hence, MMc cells in one in two embryos would be enriched using magnetic cell sorting. (**b**) Migration of MMc cells are reported to starts at around mouse embryonic stage E12.5–E13.5^23,24^, and the frequency of MMc cells increases as the fetal development proceeds, peaking at delivery. The number of MMc cells at the early embryonic stages was under the detection limit of the technique used in the current study. Hence, stage E14.5 embryo was used to detect MMc cells in the early migratory phase. (**c**) Overall experimental flow from whole-embryo dissection to MMc cell type estimation. E14.5 whole mouse embryo was processed using a whole-embryonic dissociation method developed previously^20^. Dissociated cell suspension from a single embryo was subsequently processed using magnetic cell sorting and flow cytometric cell sorting to isolate GFP-positive, live MMc cells, followed by scRNA-seq. MMc cell type was determined by mixed-clustering analysis with publicly available reference scRNA-seq data (TabulaMuris dataset^26^). Manual drawing, together with Adobe Photoshop (https://www.adobe.com, Photoshop 2021) were used to create the images.

**Figure 2:**
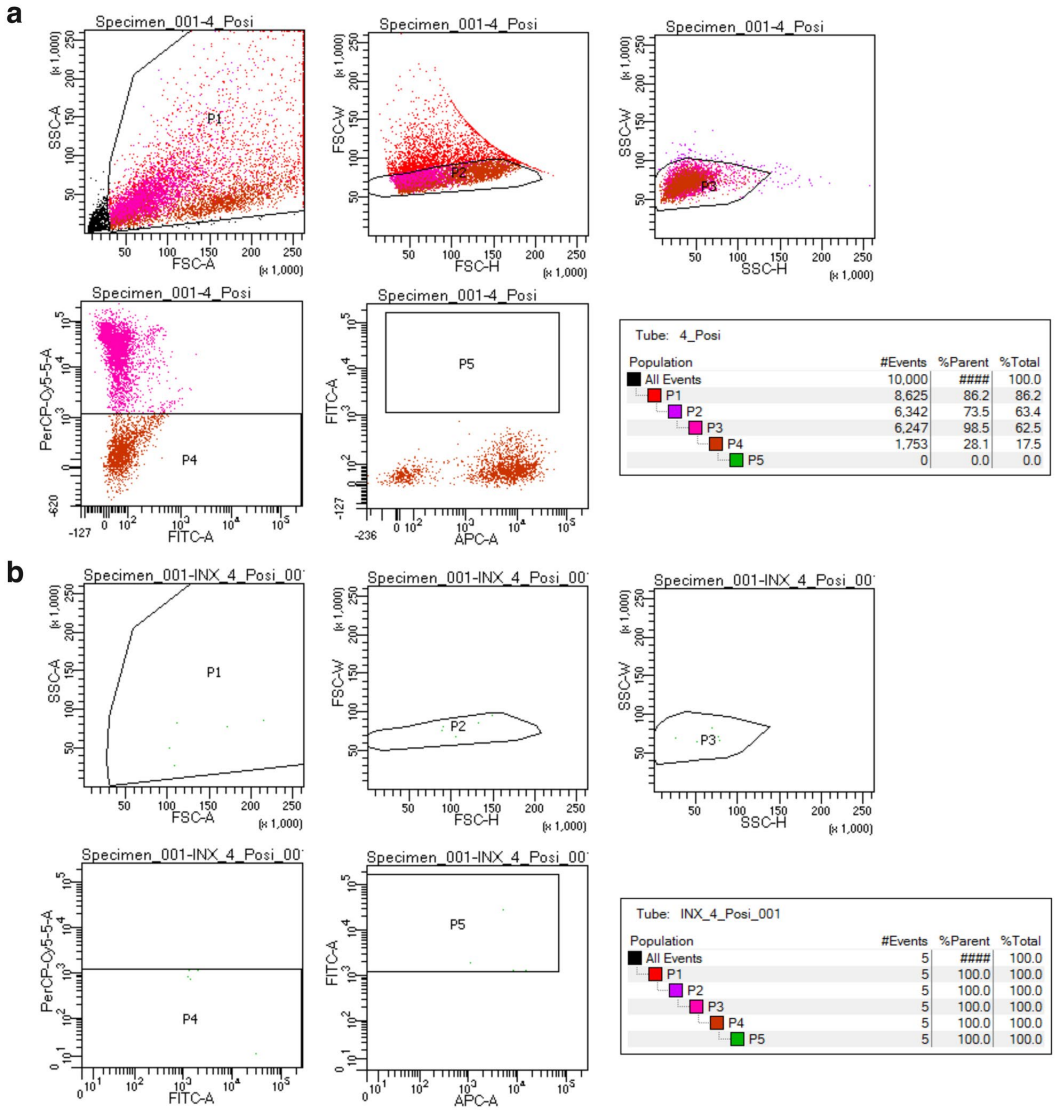
Gating conditions for sorting MMc cells. Noise and/or debris were removed with gate P1, and then doublet cells were removed with gate P2 and P3. Live-GFP positive cells (PerCP-Cy5-5 negative and FITC positive) were isolated by gate P4 and P5. (**a**), overall sorting result of a fetal cell sample. P5 gate was set for isolating MMc cells. Since all sorted cells cannot be plotted because of the counting limitation of processed cells even if the sample contains MMc cells, it will disappear from the display. (**b**) Therefore, we conducted index sorting at the same time. Panel (**b**) shows that 5 target cells were actually present and sorted. The images were created by default programs available in BD AriaIIIu (https://www.bdbiosciences.com/) and Adobe Photoshop (https://www.adobe.com, Photoshop 2021).

We utilized a transgenic mouse line that systemically expresses *GFP* (Okabe*GFP*^21^), in which MMc cells can be detected as GFP positive cells in GFP negative embryos (Fig. 1a). According to previous studies, MMc cells are detected as early as the E12.5–E13.5 mouse embryonic stage^22,23^. Further, MMc cell migration increases throughout gestation, peaking at parturition^24^. Hence, to clarify the initial cell type repertoire of the MMc cell population, and to minimize bias from estimating cell types differentiated from MMc cells with multipotent stem potential, we analyzed E14.5 embryos in the current study (Fig. 1b). To obtain MMc cells from individual embryos, the embryos were excised, washed, digested, and the cells mesh-filtered for a dissociated-cell suspension, as previously reported by us^20^ (see also “Methods” section). The cell suspension was then processed using magnetic cell sorting system to enrich MMc cells, followed by selection of live MMc cells using flow cytometric cell sorting (Fig. 2). scRNA-seq was then performed to obtain transcriptomic data for single cells. Estimation of cell types were done by combining the transcriptomic data for MMc cells with reference data from Tabula Muris database^25^, to perform mixed-clustering analysis. The experimental workflow is shown in Fig. 1c (see “Methods” for details).

For 52 GFP-negative E14.5 fetuses processed, live GFP-positive cells were detected in 26 embryos. Following preparation of the sequencing library and quality checking, 210 qualified cells were obtained and sequenced using NovaSeq6000 platform (Illumina), with an average read depth of 7,974,354 reads/cell. In each cell, 6772 genes on average were detected [transcripts per million (TPM) > 0, Supplementary Fig. 1, Additional file 1]. After removing 19 cells with no *GFP* expression detected in the RNA-seq data, a set of 191 GFP-positive cells isolated from 26 embryos was obtained. To reduce the contamination of GFP-positive cells derived from littermates within the identified 191 GFP-positive cell population, we have checked if constitutively expressed Y-linked genes are absent from these cells. Although we found no known Y chromosome-specific genes that are constitutively expressed in all cells, some genes such as zinc finger protein 1 (ENSMUSG00000053211), Y-linked, lysine (K)-specific demethylase 5D (ENSMUSG00000056673), and H2A histone family member L2B were found to be Y chromosome-specific and all of these were not expressed in the 191 GFP-positive cells. Importantly, none of the cells expressed these genes, in addition to the Y-chromosome specific *SRY* gene (ENSMUSG00000069036), suggesting that the isolated GFP-positive cells were most likely female origin. These observations suggest that the identified cells were most likely MMc cells. Nonetheless, as a caveat, it is possible that a minor proportion of these cells migrated from female GFP-positive siblings.

### Immune-related cell is the major isolated MMc cell type

**Figure 3:**
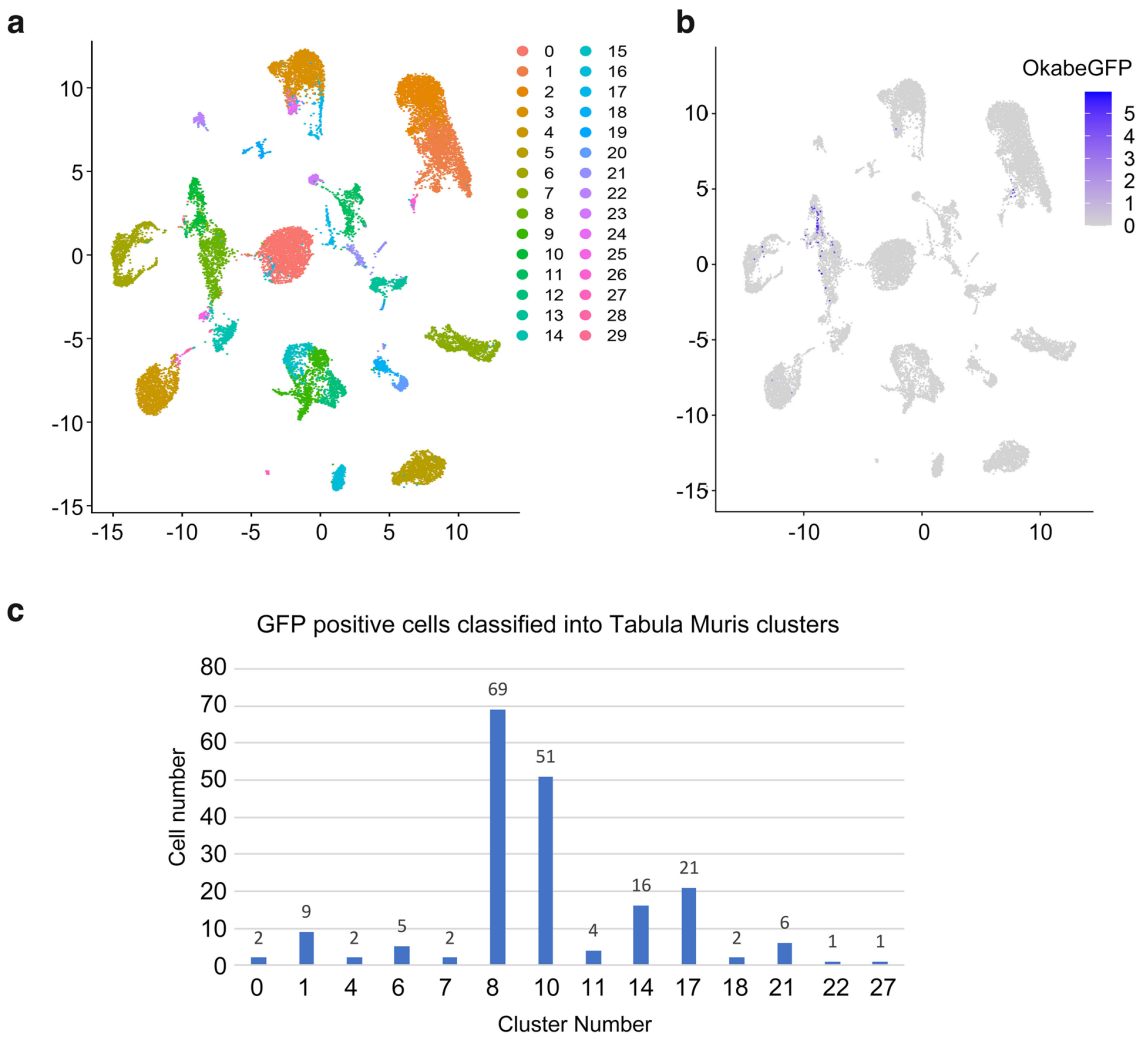
Mixed-clustering analysis of Tabula Muris data and isolated GFP-positive cells. (**a**) ScRNA-seq data of isolated cells (210 cells) was combined with Tabula Muris female data (representing 20,586 cells) and clustered by Seurat using a set of 11 principal components (see Methods). Thirty cell clusters were identified and the overall clustering pattern of the data was illustrated by using uniform manifold approximation and projection (UMAP). (**b**) In the figure, 191 Okabe*GFP*-expressing cells (as confirmed by scRNA-seq data; 19 of the isolated cells were GFP-negative) are shown as purple dots on the same UMAP plot as in (**a**). GFP-positive MMc cells were classified into multiple clusters, indicating that MMc cells represent multiple cell types. Gradation of GFP (0–5) indicates the expression of the Okabe*GFP* gene. (**c**) Bar plot representation of GFP-positive cells categorized into UMAP clusters. Majority of cells clustered into either cluster 8 (myeloid cell, 69 cells) or cluster 10 (granulocyte, 51 cells). R (https://cran.r-project.org, ver.3.6.1) with Seura package (ver. 4.0.4, https://satijalab.org/seurat/articles/pbmc3k_tutorial.html), and Microsoft EXCEL (https://www.microsoft.com, EXCEL for Mac ver. 16.62) were used to create the images and the graph.

**Figure 4:**
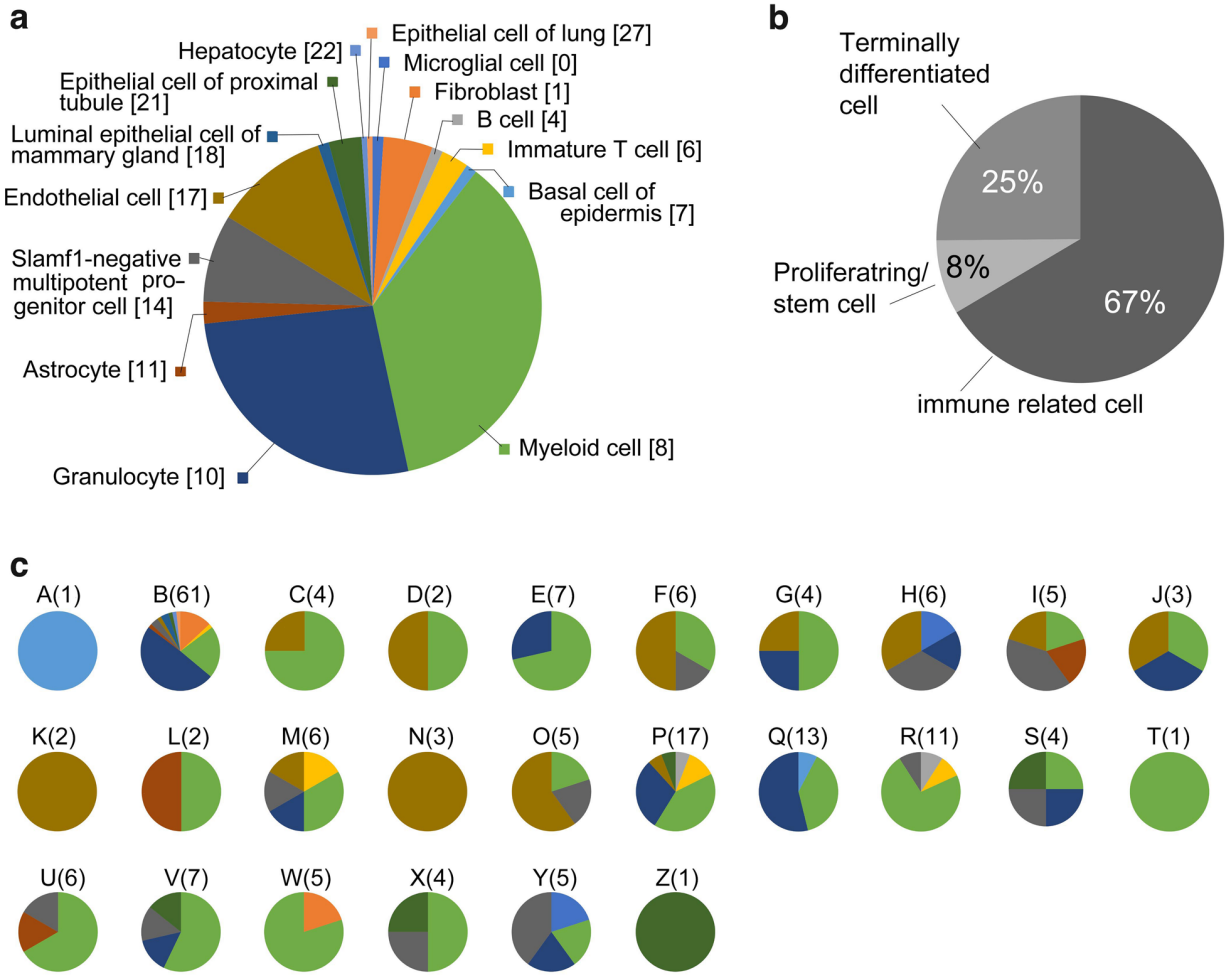
Estimation of MMc cell types. (**a**) Data for MMc cells from 26 embryos were combined (191 cells in total) and are shown as a pie-chart of the proportion of the estimated cell types. The numbers in brackets indicate cluster number after analysis using 11 PC set. (**b**) Pie-chart of the prevalence of the isolated MMc cells in three categories: immune-related cells, proliferating/stem cells, and terminally differentiated cells. (**c**) MMc cell type composition of each embryo, shown as pie-charts. Numbers in parentheses indicate the number of GFP-positive cells identified in each embryo. The color-coding is as in (**a**). Microsoft EXCEL (https://www.microsoft.com, EXCEL for Mac ver. 16.62) was used to create the images.

Before determining the type of the isolated MMc cells by clustering with the Tabula Muris dataset of female origin (20,586 scRNA-seq data for 20 organs and with 79 cell types annotated, Supplementary Fig. 2), we tested the optimal calculation parameters for classifying the Tabula Muris data. Briefly, we constructed an elbow plot representing the relationship between the explained variance and principal components (PCs) (Supplementary Fig. 3a). Two PC sets (PCs 1–11, and 1–20) were identified as candidate parameters for cell classification. While both PC sets classified the Tabula Muris cells well, cell type annotations using the 11 PC set matched the annotations in Tabula Muris better than those using the 20 PC set (Supplementary Fig. 3, Supplementary Tables 1, 2, see also “Methods”). We therefore used the 11 PC set for cell type estimation of MMc cells. The cell type for each identified cluster was defined based on the most abundant cell type defined in the annotation file for the Tabula Muris project (see “Methods”). We performed mixed-clustering of the Tabula Muris cell data and the isolated MMc cell data. The isolated GFP-positive MMc cells were classified into 14 clusters (Fig. 3a,b) rather than falling into a single MMc cluster, suggesting that the mix-clustering approach works well for MMc cell type estimation and that MMc cells represent different cell types (Figs. 3c, 4a).

Cluster 8 (myeloid cell) contained the highest number of MMc cells, and 36% of MMc cells were classified therein. The second largest cluster was cluster 10 (granulocyte), with 27% of MMc cells classified therein. In addition to these two clusters, two clusters of immune-related cell types were identified: cluster 4 (B cell) and cluster 6 (immature T cell). Consistent with the mixed-clustering analysis, we confirmed the expression of immune-related genes in scRNA-seq data for the isolated MMc cells. For example, most of the GFP-positive cells in cluster 8 (myeloid cell) indeed expressed marker genes of dendritic cell (*Itgax*^+^, *Cd24a*^+^, and *Cd68*^+^) and macrophage (*Ptprc*^+^, *H2-Eb1*^+^, *Cd86*^+^, *Selplg*^+^, *Cd14*^+^, *Cd3e*^−^, *Cd19*^−^). Of note, cells in cluster 8 also expressed *Foxp3*, encoding a major transcription factor for regulatory T cell differentiation. While none of the GFP-positive cells in cluster 10 (granulocyte) expressed a full set of marker genes of granulocyte (*Ltf*^+^, *Pglyrp1*^+^, *Lcn2*^+^, *Camp*^+^, *Mki67*^−^, *Stmn1*^−^) or those of granulocyte–monocyte progenitor cells (*Flt3*^+^, *Kit*^+^, *Mpeg1*^+^, *Itgb2*^+^, *Ahnak*^+^, *Pld4*^+^, *Cd68*^+^, *Hp*^−^), the expression of many of these genes was detected, including one cell that expressed all granulocyte–monocyte progenitor marker genes except the *Kit* gene. Further, some cells in cluster 10 (granulocyte) expressed marker genes of dendritic cell, macrophage, or monocyte (*Ly6c2*^+^, *Cx3cr1*^+^, *Cd14*^+^, *Csflr*^+^, *Mrc1*^+^), which we expected to be included in cluster 8. Among the MMc cells in cluster 10 (granulocyte), 47% (24/51 cells) expressed marker genes of invading monocyte (*Cd11b*^+^, *Csflr*^+^, *Ly6c2*^+^, *Cd14*^+^; based on the Tabula Muris reference; Supplement-Detailed Discussion of Organ Cell Types). The two GFP-positive cells in cluster 4 (B cell) indeed expressed genes characteristic for circulating B cell (*Cd79a*^+^, *Cd79b*^+^, *Cd74*^+^, *Cd19*^+^), with the expression of mature (naive) B cell-related genes (*Chchd10*^+^, *Cd79a*^+^, *Cd79b*^+^, *Cd19*^+^, *Ms4a1*^+^, *Cd74*^+^, *Mki67*^−^, *Stmn1*^−^). While none of the GFP-positive cells in cluster 6 (immature T cell) expressed a complete set of marker genes for T cell (*Ahnak*^+^, *Thy1*^+^, *Cd3e*^+^, *Cd8a*^+^), some of the cells expressed the *Cd3* gene, encoding a T-cell receptor component. Further, we have detected autoimmune regulator (*Aire*) gene expression in four cells (in clusters 14, 18, and 21). These cells showed the gene expression pattern of medullary thymic epithelial cell (*Aire*^+^, *Cldn3*^+^, *Cldn4*^+^)^26^, suggesting that the MMc cells present cell-type-specific antigens, as well as different cell-specific antigens from the mother to the fetal cells.

In addition to the immune-related cell types, we also, unexpectedly, identified MMc cells clustered in several tissue-specific and terminally differentiated cell type clusters, such as cluster 0 (microglial cell, identified as *Cx3cr1*^+^, *P2ry12*^+^, and *Tmem119*^+^; no MMc cell in this cluster expressed all these genes), cluster 1 (fibroblast, identified as *Dcn*^+^ and *Gsn*^+^; 3 out of 9 cells in the cluster expressed all these genes), cluster 7 (basal cell of the epidermis, identified as *Cd34*^+^ and *Itga6*^+^; the two MMc cells in this cluster expressed these two genes), cluster 11 (astrocyte, identified as *Aldh1l1*^+^, *Slc1a3*^+^, and *Aqp4*^+^; no MMc cells in this cluster expressed all these genes; “blank” [defined by the Tabula Muris annotation file] cell type was the largest in this cluster), cluster 17 (endothelial cell, identified as *Pecam1*^+^; 4 out of 21 MMc cells in this cluster expressed this gene), cluster 18 (luminal epithelial cell of the mammary gland, identified as *Krt8*^+^, *Krt18*^+^, and *Krt19*^+^; the two MMc cells in this cluster expressed these genes), cluster 21 (epithelial cell of the proximal tubule, identified as *Vil1*^+^; 2 out of 6 MMc cells in this cluster expressed this gene), cluster 22 (hepatocyte, identified as *Alb*^+^, *Ttr*^+^, *Apoal*^+^, and *Serpina1c*^+^), and cluster 27 (epithelial cell of the lung, identified as *Pecam1*^−^ and *EpCAM*^+^; the one cell in this cluster did not express the marker gene). Apart from immune-related or differentiated cell types, 16 cells were classified in cluster 14 (multipotent progenitor cell), suggesting that some MMc cells have stem cell-like cell phenotype, as has been reported previously^8,18^. This was also supported by the detection of the expression of marker genes of hematopoietic stem cell (*Kit*^+^, *Stmn1*^+^, *Mki67*^+^) in 6 out of 12 cells, and a marker of cell proliferation, namely, *Mki67,* in 10 out of 12 cells in the cluster 14.

Although the number of identified MMc cells in different embryos varied widely, the presented results suggest that most MMc cells are immune cells, with the remaining cells either proliferating/stem cells, or terminally differentiated cells (Fig. 4b). Further, while a relatively small proportion of maternal stem cells was identified among the analyzed MMc cells (8% in Fig. 4b), frequent detection of these cells in GFP-positive embryos (46% of embryos, Table 1) suggests that different MMc cell types are characterized by different migration ability.

**Table 1:** MMc cell types detected in the analyzed embryos in the current study.

% of migrated individual	Cell type	# of cluster
81% (21/26 fetus)	Myeloid cell	8
50% (13/26 fetus)	Endothelial cell	17
46% (12/26 fetus)	Slamf1-negative multipotent progenitor cell	14
42% (11/26 fetus)	Granulocyte	10
23% (6/26 fetus)	Epithelial cell of proximal tubule	21
8% (2/26 fetus)	Immature T cell	6
	Astrocyte	11
8% (2/26 fetus)	Microglial cell	0
	Fibroblast	1
	B cell	4
	Basal cell of epidermis	7
4% (1/26 fetus)	Luminal epithelial cell of mammary gland	18
	Hepatocyte	22
	Epithelial cell of lung	27

### Proportions of immune-related and stem cell-like MMc cells differ in individual embryos

While MMc cells are thought to be present in all individuals, including in healthy and diseased individuals, association of an increased occurrence of MMc with a variety of physiological phenomena (including tolerance^5–8^, regeneration^4,9–11^, and fetal tissue damage or enhancement of certain congenital diseases) has been reported^12–16^. These variable outcomes could be caused by environmental factors, such as pathogens^19^, and/or major histocompatibility complex (MHC) compatibility^14^ between the mother and the fetus; however, they might also be explained by differences in the MMc cell type repertoire between individuals. In our previous study, we showed that the frequency of MMc cells largely differs among inter-individual embryos^20^. However, the existence of inter-individual variations in MMc cell types remained to be clarified. Accordingly, in the current study, data for the MMc cells isolated from 26 embryos implied that the proportions of MMc cell types differ between individuals (Fig. 4c; Supplementary Table 3). Since the potential role of MMc cells in the fetus and the neonate is often related to the immune system, such as immunological tolerance, with activation of cytotoxic profile upon depletion (Castellan et al*.*, submitted), we also analyzed the potential variation in the proportion of immune-related MMc cells (defined by clustering analysis with Tabula Muris data) in different embryos. We found statistically significant differences in the proportion of immune-related MMc cells among individual embryos (Table 3, p = 4.95 × 10^−6^, Fisher’s exact test, two-sided). Similarly, we detected a significant variation in the proportion of MMc proliferating/stem cells (Table 2, p = 0.0398, by Fisher’s exact test, two-sided). Taken together, these observations suggest that the MMc cell type repertoire differs between embryos.

**Table 2:** The proportions of proliferating/stem MMc cells (clusters 14) and the other cell types in individual embryos.

Individual ID	Proliferating/stem cell types	The other cell types
A	0	1
B	2	59
C	0	4
D	0	2
E	1	7
F	0	5
G	2	4
H	2	4
I	0	3
J	0	3
K	0	2
L	1	2
M	0	5
N	0	3
O	1	4
P	0	17
Q	0	13
R	1	10
S	1	3
T	0	1
U	1	5
V	1	6
W	0	5
X	1	3
Y	2	3
Z	0	1

The proportions of the proliferating/stem MMc cells were significantly different among the embryos (p-value = 0.03976, Fisher’s test, two-sided).

### Most MMc cells express migration-related proteins

To shed light on the mechanism underpinning MMc cell migration from the mother to the fetus, we searched for genes that were commonly expressed in MMc cells (genes with TPM > 1, and detected in more than 90% of MMc cells). After excluding housekeeping genes, such as ribosomal protein genes, we identified a few genes that could be involved in MMc cell migration. One of them was interferon-induced transmembrane protein 2 (*Ifitm2*) gene, encoding a member of the interferon-induced transmembrane (IFITM) family. While studies focusing on this gene are scarce, *Ifitm3*, encoding another member of this family, is expressed in migratory primordial germ cells, and reportedly regulates cell adhesion and differentiation^27^. We also identified syndecan-binding protein (*Sdcbp*) gene, known to be involved in cell migration and invasion of tumor metastasis in human^28^. As a third candidate, we detected a gene for macrophage migration inhibitory factor (MIF), an important mediator of the innate immune system^29^. Although further functional studies are required, these results imply that these gene could be involved in the migration of MMc cells.

## Discussion

In the present study, we estimated the MMc cell type repertoire of individual embryos and demonstrated that different embryos have different MMc type repertoires. These findings provide a potential hint toward the mechanism underlying the previously reported associations between cells of maternal origin and different physiological phenomena in the progeny^4–16^.

In pioneering studies on MMc cells, their cell types have been often determined using immunostaining with a limited set of antibodies, which hindered comprehensive analysis of MMc cell types^3,30^. Further, previous studies tended to focus on immune-related cells^17,31,32^, since MMc cells are non-self to the fetus (especially those expressing non-inherited maternal antigens), which implies that certain immunological reconciliation is required between the mother and the fetus. In the current study, by utilizing our previously established MMc isolation method and combining it with scRNA-seq (Figs. 1,2), we successfully determined the cell type of 191 potential MMc cells isolated from 26 whole mouse embryos (Figs. 3,4). However, since we took advantage of MHC to minimize the contamination of non-maternal cells, it must be noted that some rare stem cells that do not express MHC could have been overlooked.

We showed here that the majority (67%) of the isolated MMc cells represent immune-related cell types, mainly myeloid cell and granulocyte, rather than B cell and T cell. While the proportion of maternal proliferating/stem cells was relatively small in the overall MMc cell population (8%, Fig. 4b), almost half (46%) of the analyzed fetuses contained this cell type (Table 1), suggesting a high migratory potential of stem cells, at least toward the fetus. As the number of MMc cells increases toward delivery^24^, these maternal proliferating/stem cells are reasonably assumed to create a stem cell niche in the fetus, contributing to the long-term existence of maternal cells in the offspring after birth^4^.

Of note, the proportions of the immune-related cells differed significantly among the individual embryos (Table 3), and we observed a similar tendency for stem cell-like MMc cells expressing proliferation marker genes. Although further studies are needed to confirm the biological importance, these findings imply that the inter-individual variability of MMc cell type repertoire could underpin the different physiological phenomena associated with MMc cells (immunological tolerance, regeneration, and cause or deterioration of congenital diseases).

**Table 3:** The proportions of immune-related MMc cells (clusters 4, 6, 8, 10) and the other cell types in individual embryos.

Individual ID	Immune-related cell types	The other cell types
A	0	1
B	44	17
C	3	1
D	1	1
E	7	0
F	2	4
G	3	1
H	1	5
I	1	4
J	2	1
K	0	2
L	1	1
M	4	2
N	0	3
O	1	4
P	15	2
Q	12	1
R	10	1
S	2	2
T	1	0
U	4	2
V	5	2
W	4	1
X	2	2
Y	2	3
Z	0	1

The proportions of immune-related MMc cells were significantly different among the embryos (p-value = 4.946 × 10^−6^, Fisher’s test, two-sided).

Terminally differentiated tissue-specific cell types, such as hepatocyte, astrocyte, and epithelial cells (Fig. 4a,b), roughly accounted for 25% of the identified MMc cells. Although we aimed to capture the early-migratory phase MMc cells, it is nonetheless possible that these terminally differentiated MMc cells arose from division and differentiation of stem cell-like MMc cells that we could not detect in our dataset. However, irrespective of the lineage, these findings indicate that the embryo comes in contact with a variety of non-inherited maternal antigens via differentiated MMc cells, which could contribute to the immunological tolerance or anergy against non-inherited maternal antigens. Consistently, in addition to dendritic MMc cells, we detected some cells expressing the *Aire* gene, which drives the expression of tissue-restricted antigen genes to induce tolerance against these antigens^33^. Although a reduced fetal CD8^+^ T-cell population has been reported in mice lacking maternal T and B cells^32^, maternal immune cells other than T and B cells may play an important role in the development of the fetal immune system. In fact, a recent pioneering study demonstrated that MMc cell numbers and their microchimerism-derived extracellular vesicles modify the fetal immune system^34^. These findings further highlight the importance of non-T and non-B maternal cells, such as maternal dendritic cells, in the development of the fetal immune system. Apart from the well-known functions of dendritic cells and macrophages^35^, it is possible that they contribute to regulation of fetal immune system via an unknown mechanism, as we detected *Foxp3* expression in some of these cells. This observation was consistent with a report that *Foxp3* is expressed in macrophages infiltrating the kidney in renal cancer^36^; however, the immuno-suppressive ability of these cell types remains to be clarified. Further, the identification of differentiated cells in the early developmental phase implies a possible and direct contribution of terminally differentiated maternal cells to fetal tissues, rather than their differentiation from undifferentiated MMc stem cells. The remaining (roughly 8%) of MMc cells expressed the genes of proliferating or multipotent progenitor cells, which is in agreement with a report that MMc cells last in the offspring for decades after birth^4^.

Taken together, the current study provides fundamental information for understanding MMc cells, and might explain the possible individual bias toward the variety of physiological phenomena related to these cells^4–9,11–16^. As the genetic variation of humans is much higher than that of inbred mice used in the current study, it is likely that cell type variations and the MMc cell-related bias toward the different phenomena in humans are greater than those described herein. On the other hand, as a caveat of the current study, the cell types were here estimated based on gene expression rather than protein levels, and hence further studies are needed to confirm the identification and variability of the MMc cell type repertoire. Similarly, the sensitivity of different MMc cell types to experimental procedures could differ, leading to a possible detection bias of the cell type repertoire. To add, why some of the cells did not show a full set of marker genes remains unclear. While read-depth per cell could have been a bit too shallow to detect these genes, another possibility would be that these cells are in the transitional phase of differentiation into other specific cell types. Future studies with additional developmental stages would be helpful to solve this problem. Finally, although we have succeeded in near-comprehensive estimation of MMc cell types, the exact mechanisms behind the migration and differentiation of MMc cells awaits further study. Nonetheless, we were able to detect the expression of migration-related genes in the majority of isolated MMc cells (> 90%). Examples include *Ifitm2* gene, a paralog of *Ifitm3*. The latter is expressed in migratory primordial germ cells (PGC), regulates cell adhesion and differentiation^27^, and its product co-localizes with Ifitm2 in the intracellular space^37^. Further, *Ifitm3* overexpression promotes the metastasis of hepatocellular carcinoma^38^. Based on the fact that we found no PGC cells in our dataset, it is possible that MMc cells utilize these cell migration-related proteins (e.g., Ifitm2, SDCBP, and MIF^29^) to migrate from the mother to the fetus. Further study of these genes with respect to migration into the fetus could provide additional information for understanding MMc cell migration.

## Conclusions

While some MMc cell types have been previously described, the comprehensive identification of MMc cell types remained to be clarified. In the current study, by using our previously established MMc cell-isolation technique in conjunction with scRNA-seq, we, for the first time, provide a comprehensive overview of the MMc cell type repertoire. The majority of the isolated MMc cells were immune-related cells, terminally differentiated cells, and stem cell-like proliferating cells. We also found that the proportion of immune-related cells and proliferating stem cells of maternal origin significantly differs among individual embryos. These findings provide not only a basis for the understanding of various MMc-related phenomena, but also provides insight into what is actually inherited to offspring by cells of maternal origin.

## Methods

### Mouse mating strategy for GFP^+^H-2 Kb^+^ labeling of MMc cells

BALB/cByJJcl inbred strain was obtained from Clea Japan. *GFP*-expressing mice, C57BL/6-Tg(CAG-EGFP)C14-Y01-FM131Osb, developed by Okabe et al.^21^, were from RIKEN BioResource Research Center (RBRC). Genotyping was performed according to the instructions provided by RBRC using polymerase chain reaction (PCR). To obtain wild-type fetuses with GFP-positive MMc cells, *GFP*-heterozygous female mother mice were mated with BALB/cByJJcl, GFP^−/−^ male mice, and only fetuses lacking the *GFP* gene (n = 52) were used in subsequent experiments (Fig. 1) in detecting GFP^+/−^ maternal cells. Furthermore, to prevent detection of GFP-positive grand-maternal cells^39^, the mother mice were obtained by crossing BALB/cByJJcl wild-type female mice with *GFP*-homozygous male mice. MHC types of mice were carefully designed for utility to concentrate MMc cells using magnetic cell sorting system (Fig. 2).

### Tissue dissociation for single-cell isolation from a whole embryo

Single-cell suspensions collected from the wild-type E14.5 fetus containing GFP-positive MMc cells were obtained as previously described^20^. Stage E14.5 embryo was used for cell type estimations of the early migrating population of MMc cells, as E12.5–E13.5 is the earliest phase of their migration^22,23^. Briefly, the GFP-negative embryo was identified by the absence of fluorescence using a GFP-excitation flashlight while the embryo was in the amnion. The mother mouse was then sacrificed by cervical dislocation, and the embryo was carefully dissected and washed to avoid cross-contamination of fetal and maternal cells. In this process, we first cut out maternal blood vessels surrounding the uterus at the closest point to the uterus to avoid carrying maternal blood to the next step. After washing the uterus with embryos, we repeated the washing process with a new Phosphate buffered Saline (PBS), and then cut open the uterus with scissors. Embryos were then dissected out with amniotic membranes untouched, and only embryos without fluorescence (checked with UV light) were moved into a new PBS and washed. The amniotic membrane was then cut open with new tweezers, and the umbilical cord was cut at the embryonic side while squeezing it with tweezers to avoid losing readily migrated MMc cells. While keeping the umbilical cord squeezed with tweezers, an embryo was washed three times with new PBS in new dishes, and then dried by wiping off PBS with kimwipes and kimtowels. Then, the embryo was minced, the material filtered twice using 100-µm and 70-µm cell strainers, and blood cells removed by ammonium-chloride-potassium (ACK) lysis buffer treatment to obtain a single-cell suspension. Cell density in each suspension was determined using a hemocytometer, and the antibody volume was measured based on this for use of magnetic cell sorting. The blood of the sacrificed mother was used as a positive control, while that of wild-type mouse was used as a negative control. For the control blood cells, erythrocytes were removed as in the fetus samples, followed by ACK treatment for 3 min.

### MMc cell enrichment by magnetic cell sorting

Before analyzing the single-cell suspensions by flow cytometric cell sorting, MMc cells were enriched using magnetic cell sorting (Miltenyi Biotec K.K.). This was done by taking advantage of the differential expression of H-2 Kb proteins on maternal and fetal cells, as *GFP*-heterozygous mother mice express H-2 Kb protein as MHC on the cell surface, while only one in two fetuses, by chance, expresses H-2 Kb protein. Single-cell suspensions from fetuses were stained by allophycocyanin (APC)-conjugated anti-mouse H-2 Kb antibody (clone AF6-88.5, BioLegend, cat. no. 116518), followed by mixing with anti-APC beads (Miltenyi Biotec K.K., cat. no. 130-090-855), and then concentrated by following the protocol of the magnetic cell sorting manufacturer. Cell density was determined by hemocytometer, and cell samples were centrifuged at 300×*g* for 10 min at 4 ℃. After removing the supernatant, the supernatant of hybridoma 2.4G2 was added as an Fc receptor blocker, and the samples incubated for 10 min at 4 ℃. Then, APC anti-mouse H-2 Kb antibody (clone AF6-88.5, BioLegend, cat. no. 116518) was diluted 1:80 in magnetic cell sorting buffer [D-PBS, 0.2% (v/v) BSA], added to blocked cell samples, and incubated for 30 min at 4 ℃ in the dark. To remove excess antibody, flow cytometric cell sorting buffer [Hank’s balanced salt solution (HBSS) (−), 0.5% (w/v) BSA, 2 mM EDTA] was added and the samples centrifuged at 330×*g* for 6 min at 4 ℃. Anti-APC MicroBeads (Miltenyi Biotec K.K., cat. no. 130-090-855) diluted into 1:80 as the final concentration were then added to cell suspensions, pipette-mixed, and incubated for 15 min at 4 ℃ in the dark. Next, the magnetic cell sorting buffer was added to remove excess beads, followed by centrifugation at 330×*g* for 6 min at 4 ℃. After removing the supernatant, 500 μl of magnetic cell sorting buffer was added to the cell suspensions, and the samples were loaded onto an equilibrated magnetic column (MS Column, Miltenyi Biotec K.K., cat. no. 130-042-201) on a magnet stand (OctoMACS Separator, Miltenyi Biotec K.K., cat. no. 130-042-109), after passing through a 70-μm mesh filter (the column was equilibrated with 500 μl of magnetic cell sorting buffer before sample loading). The loaded column was then washed three times with 500 μl of magnetic cell sorting buffer. After washing, the column was removed from the magnetic field. Then, 1 ml of magnetic cell sorting buffer was loaded, the target cells were pushed out using a syringe, and effluent fractions containing target cells were collected. The collected cell fractions were centrifuged at 330×*g* for 6 min at 4 ℃. The supernatant was removed by adding 100 μl of magnetic cell sorting buffer to obtain the positive fractions with the target cells and 200 μl of the buffer to the negative fractions without the target cells. As a positive control, maternal blood cells were stained using the same antibody as the fetal cells to confirm the specificity of immunostaining conditions and to determine the gating conditions of flow cytometric cell sorting for MMc cell isolation. The immunostaining protocol of the maternal cells were the same as that for fetal cell immunostaining. For the unstained cells, an equivalent volume of magnetic cell sorting buffer was added, instead of the antibody solution. Finally, after removing excess antibody and centrifugation under the same condition as fetal cells, the cells were suspended in 100 μl of magnetic cell sorting buffer.

### MMc isolation by flow cytometric cell sorting

Before analyzing cell suspensions by flow cytometric cell sorting (BD AriaIIIu), all samples were stained with propidium iodide (PI) solution (diluted into 1:1000 as the final concentration) to separate live cells from dead cells. Maternal blood cells and wild-type blood cells were used for setting the appropriate gating conditions. For the experiment, MMc cells were isolated as PI^−^GFP^+^APC^+^ cells (Fig. 2). MMc cells were collected using single-cell sorting mode, and sorted into a 96-well PCR plate or 8-strip tubes in 3 μl of receiving solution. The receiving solution was prepared by mixing 0.5 μl of 10× reaction buffer [prepared using 10× lysis buffer containing 0.05% (v/v) of RNase inhibitor (both reagents were from SMART-Seq v4 Ultra Low Input RNA Kit for Sequencing, Clontech, cat. no. 634890)] and 2.5 μl of RNase-free water. The collected cells were centrifuged and stored at − 80 ℃ until sequencing.

### ScRNA-seq of MMc cells

For scRNA-seq of MMc cells, Smart-seq2^40^ protocol was adopted. By following the User Manual of SMART-Seq v4 Ultra Low Input RNA Kit for Sequencing, cDNA synthesis and its amplification by LD-PCR was done. Amplified nucleic acids were purified using Agencourt AMPure XP kit (Beckman Coulter, cat. no. A63881) and 80% (v/v) ethanol, and sample concentration was determined using Qubit dsDNA HS Assay Kit (Invitrogen, cat. no. Q32851). Tagmentation was done using Nextera XT Library Prep Kit (96 samples, Illumina, FC-131-1096) and Nextera XT index Kit v2 set A (96 indices, 384 samples, Illumina, FC-131-2001) using a standard protocol. The concentration of multiplexed sequencing libraries was adjusted to 10 nM and the libraries were sequenced using NovaSeq6000 platform. Embryos A and B were sequenced as 100 bp paired-end reads, whereas embryos C–Z were sequenced as 150 bp paired-end reads (for more details, see associated raw RNA-seq files publicly available in DRA database, BioProject ID: PRJDB12985).

### First-strand cDNA synthesis and amplification by LD-PCR

cDNA synthesis and amplification by LD-PCR were done following the User Manual of SMART-Seq v4 Ultra Low Input RNA Kit for Sequencing (Clontech, 634890), with some modifications under an open and clean workstation. Briefly for cDNA synthesis, the control total RNA (1 μg/μl in original concentration) was diluted to 4 × 10^−6^ μg/μl, and 3 μl of this was used to roughly match the RNA amount of a single cell (0.1 pg). In addition, 1 μl of external RNA controls consortium (ERCC) RNA Spike-In Mix (Invitrogen, cat. no. 4456740) as spike-in control was also added to all the samples and controls, for the final concentration of approximately 10,386 molecules/μl. This was followed by LD-PCR, as per manufacturer’s instructions.

### Determination of gene expression levels in MMc cells and Tabula Muris data analysis

The quality of scRNA-seq fastq files (from isolated MMc cells and Tabula Muris data) was checked by using FastQC (v0.11.9), followed by adapter trimming using Trimmomatic (v 0.39). Tabula Muris data were downloaded from Amazon Web Services (AWS) (s3://czbiohub-tabula- muris/facs_bam_files/) and transformed into fastq files by bam2fastq in samtools (v 1.34). Clean fastq files were then mapped to the mouse genome (Mus_musculus.GRCm39.dna_rm.toplevel.fa, downloaded from ensesmble database ver. 104) and EGFP sequence [extracted from EGFP sequence of pEGFP-C1 (addgene), or Okabe*GFP*] using HISAT2 (2.2.1) software. Relative gene expression level was determined by processing the mapped data using stringTie (v2.1.6) and GTF files incorporating Okabe*GFP* sequence (Mus_musculus.GRCm39.104.gff3). Only samples with more than 500 genes expressed (TPM > 0) were used for the ensuing analyses (20,586 out of 24,259 cell data from Tabula Muris and all isolated potential MMc cells passed this threshold). To estimate the type of isolated cells, Tabula Muris data, which consists of massive scRNA-seq data for 20 organs^25^ were used as a reference. Only Tabula Muris data from female mice were considered, as only maternal cells were targeted in the current study.

### MMc cell type estimation by mixed-clustering analysis

MMc cell type was estimated by mixed clustering using isolated MMc cell and Tabula Muris gene expression data using Seurat (ver 4.0.4). By following the Seurat‒Guided Clustering Tutorial (compiled: August 30, 2021), linear dimensional reduction (principal component (PC) analysis) was first performed to determine the dimensionality of the Tabula Muris dataset. Elbow plot was adopted for dimensionality determination. Presence of several elbows in the plot (Supplementary Fig. 3a) suggested that the majority of variance was likely to be captured by the first 2, 6, 11, or 20 PCs. Cell types in each cluster were defined according to the major cell type (defined by annotations_facs.csv provided in the Tabula Muris project) that constituted the cluster. Based on the results, a uniform manifold approximation and projection (UMAP) plot was created for these PC numbers (Supplementary Fig. 3b). For the analysis, 11 PC and 20 PC sets were used, as these resulted in relatively high clustering resolution. To determine which PC set (11 PCs or 20 PCs) was suitable for the ensuing analysis, clustering of Okabe*GFP*-expressing cells, or MMc cells, in multiple clusters for the two PC sets was first verified, suggesting that Tabula Muris can be utilized for cell type estimation of MMc cells. For the two PC sets, the cell type of each cluster was defined based on the highest number of cells (Supplementary Tables 2, 3), and the presence of a unique cell type in each cluster was evaluated. For the 11 PC set, two cell types were clustered into multiple clusters (endothelial cell in clusters 3 and 17, and luminal epithelial cell of mammary gland in clusters 16 and 18). For the 20 PC set, three cell types were clustered into multiple clusters (endothelial cell in clusters 2 and 23, immature T cell in clusters 10 and 18, and luminal epithelial cell of mammary gland in clusters 14 and 19). Considering the number of cell types clustered into multiple clusters, the 11 PC set performed better than the 20 PC set and was used in the ensuing analyses. In some types of cells, final confirmation of the cell types was done by checking the expression of cell-type specific marker gene expressions of GFP positive MMc cells based on this clustering result with 11 PCs.

### Ethics approval and consent to participate

The study was carried out in compliance with the ARRIVE guidelines. All of the animal care was conducted in strict accordance with the relevant guidelines and regulations (Animal Plan ID: 03-05). The study was approved by the animal science committee in the School of Science, the University of Tokyo (approval ID: 17-2). All efforts were made to minimize suffering. Pregnant female mice were first anesthetized by exposure to isoflurane and next euthanized by cervical dislocation.

## Supplementary Information

Supplementary Figures.

Supplementary Tables.

## Data Availability

RNA-seq files are publicly available in DRA database (https://www.ddbj.nig.ac.jp/) with BioProject ID: PRJDB12985.

## Abbreviations

APC: Allophycocyanin
AWS: Amazon web services
GFP: Green fluorescent protein
HBSS: Hank’s balanced salt solution
MHC: Major histocompatibility complex
MMc: Maternal microchimeric
PC: Principal component
PCR: Polymerase chain reaction
scRNA-seq: Single-cell RNA sequencing
TPM: Transcript per million
UMAP: Uniform manifold approximation and projection
