# Supplementary material for: Whole-embryonic identification of maternal microchimeric cell types in mouse using single-cell RNA sequencing
Source: Sci Rep. 2022 Nov 4;12:18313. doi: 10.1038/s41598-022-20781-9 (PMC9636240; doi:10.1038/s41598-022-20781-9)
Supplement: Supplementary file 1 — Supplementary Figures. [file 41598_2022_20781_MOESM1_ESM.pdf]

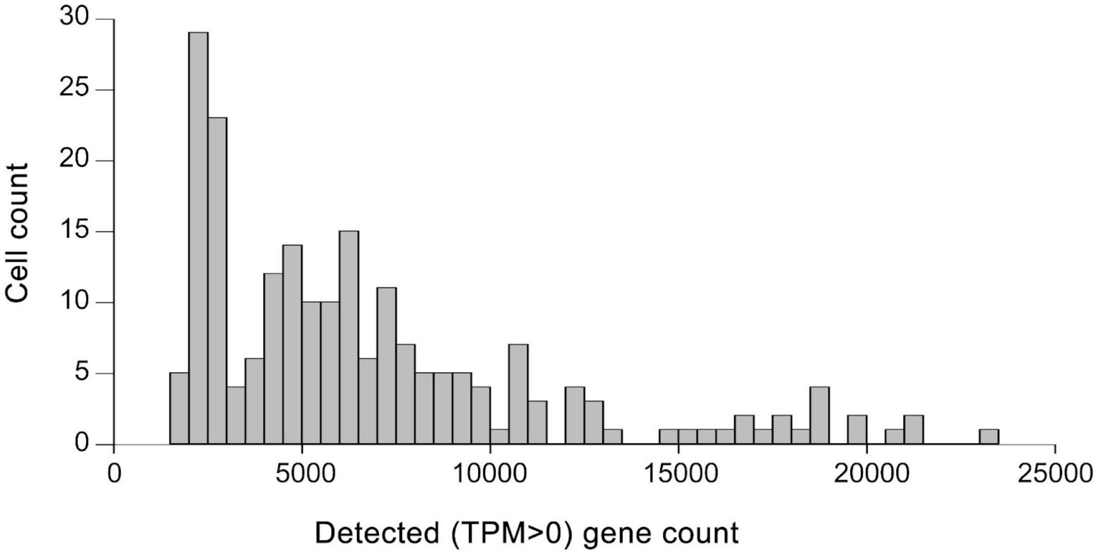

Supplementary Figure 1 Gene count in sequenced cells. The number of genes expressed (TPM>0) in isolated sequenced cells (MMc cells) was determined by analyzing scRNA-seq data. R (https://cran.r-project.org, ver.3.6.1) was used to create the barplot.

a

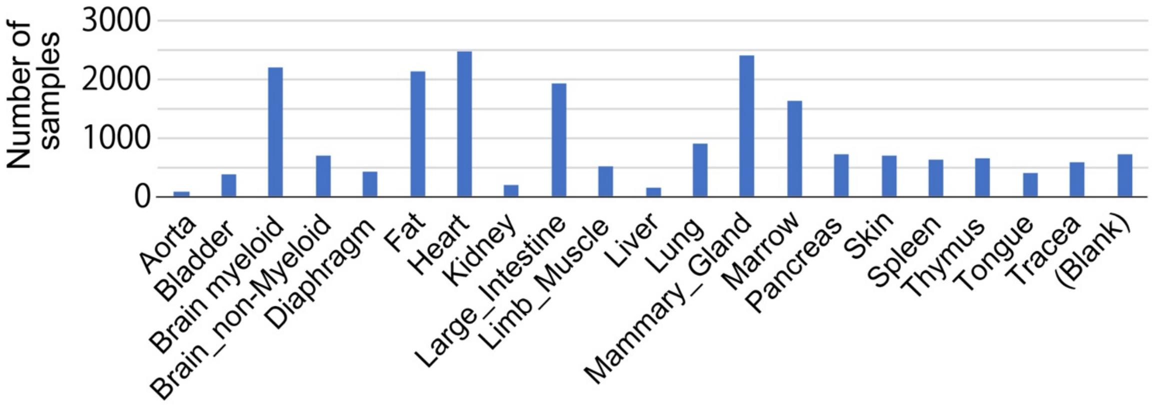

b

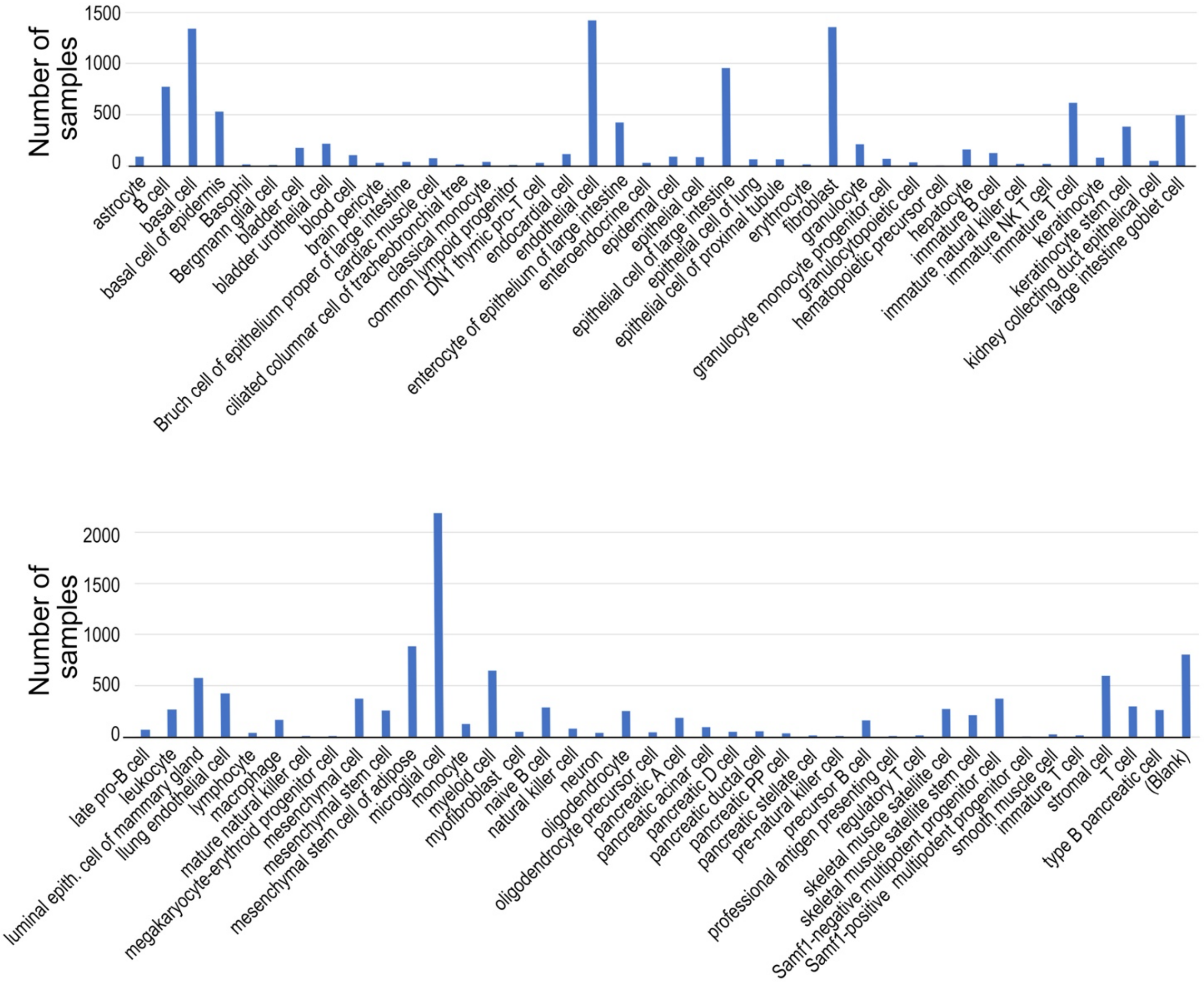

Supplementary Figure 2 Organs and cells included in the Tabula Muris data for female mice. (**a**) Organs represented in the data and their sample (cell) number. (**b**) Cell types represented in the data and their sample (cell) number. R (https://cran.r-project.org, ver.3.6.1) was used to create the graphs.

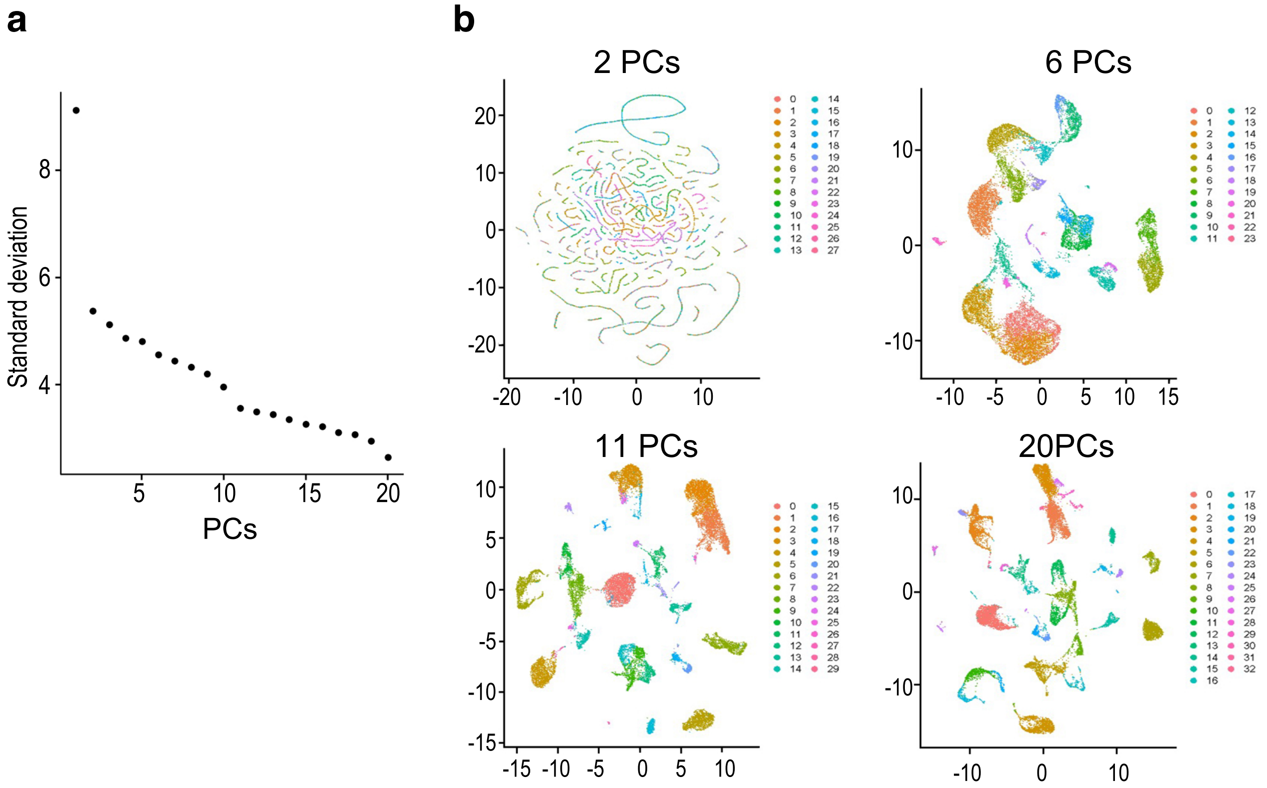

Supplementary Figure 3 Identification of optimal PC sets for classifying the Tabula Muris scRNA-seq data. (**a**) Elbow plot representing the relationship between standard deviation and the number of PCs used in calculation. A major change in standard deviation was observed with 2, 6, 11, and 20 PCs. (**b**) UMAP representation of Tabula Muris scRNA-seq data using the PC sets identified in (**a**). R (https://cran.r-project.org, ver.3.6.1) was used to create figures.
