# Supplementary material for: Whole-embryonic identification of maternal microchimeric cell types in mouse using single-cell RNA sequencing
Source: Sci Rep. 2022 Nov 4;12:18313. doi: 10.1038/s41598-022-20781-9 (PMC9636240; doi:10.1038/s41598-022-20781-9)

**Supplementary Table 1** Major cell types of clusters from UMAP analysis based on 11 PCs

| **Cluster #** | **Cell type** |
| --- | --- |
| 0 | microglial cell |
| 1 | fibroblast |
| 2 | mesenchymal stem cell of adipose |
| 3 | endothelial cell |
| 4 | B cell |
| 5 | basal cell |
| 6 | immature T cell |
| 7 | basal cell of epidermis |
| 8 | myeloid cell |
| 9 | epithelial cell of large intestine |
| 10 | granulocyte |
| 11 | astrocyte |
| 12 | large intestine goblet cell |
| 13 | type B pancreatic cell |
| 14 | Slamf1-negative multipotent progenitor cell |
| 15 | enterocyte of epithelium of large intestine |
| 16 | luminal epithelial cell of mammary gland |
| 17 | endothelial cell |
| 18 | luminal epithelial cell of mammary gland |
| 19 | pancreatic acinar cell |
| 20 | bladder urothelial cell |
| 21 | epithelial cell of proximal tubule |
| 22 | hepatocyte |
| 23 | oligodendrocyte |
| 24 | endocardial cell |
| 25 | macrophage |
| 26 | brain pericyte |
| 27 | epithelial cell of lung |
| 28 | late pro-B cell |
| 29 | granulocytopoietic cell |

**Supplementary Table 2** Major cell types of clusters from UMAP analysis based on 20 PCs

| **Cluster #** | **Cell type** |
| --- | --- |
| 0 | microglial cell |
| 1 | fibroblast |
| 2 | endothelial cell |
| 3 | mesenchymal stem cell of adipose |
| 4 | B cell |
| 5 | myeloid cell |
| 6 | basal cell |
| 7 | keratinocyte stem cell |
| 8 | epithelial cell of large intestine |
| 9 | Slamf1-negative multipotent progenitor cell |
| 10 | immature T cell |
| 11 | enterocyte of epithelium of large intestine |
| 12 | large intestine goblet cell |
| 13 | astrocyte |
| 14 | luminal epithelial cell of mammary gland |
| 15 | basal cell of epidermis |
| 16 | epithelial cell of proximal tubule |
| 17 | granulocyte |
| 18 | immature T cell |
| 19 | luminal epithelial cell of mammary gland |
| 20 | natural killer cell |
| 21 | pancreatic A cell |
| 22 | type B pancreatic cell |
| 23 | endothelial cell |
| 24 | bladder urothelial cell |
| 25 | pancreatic acinar cell |
| 26 | bladder cell |
| 27 | hepatocyte |
| 28 | oligodendrocyte |
| 29 | mesenchymal cell |
| 30 | myofibroblast cell |
| 31 | stromal cell |
| 32 | cardiac muscle cell |

**Supplementary Table 3** MMc cell counts and their determined cell types (numbers in brackets represent clusters after 11 PC UMAP analysis)


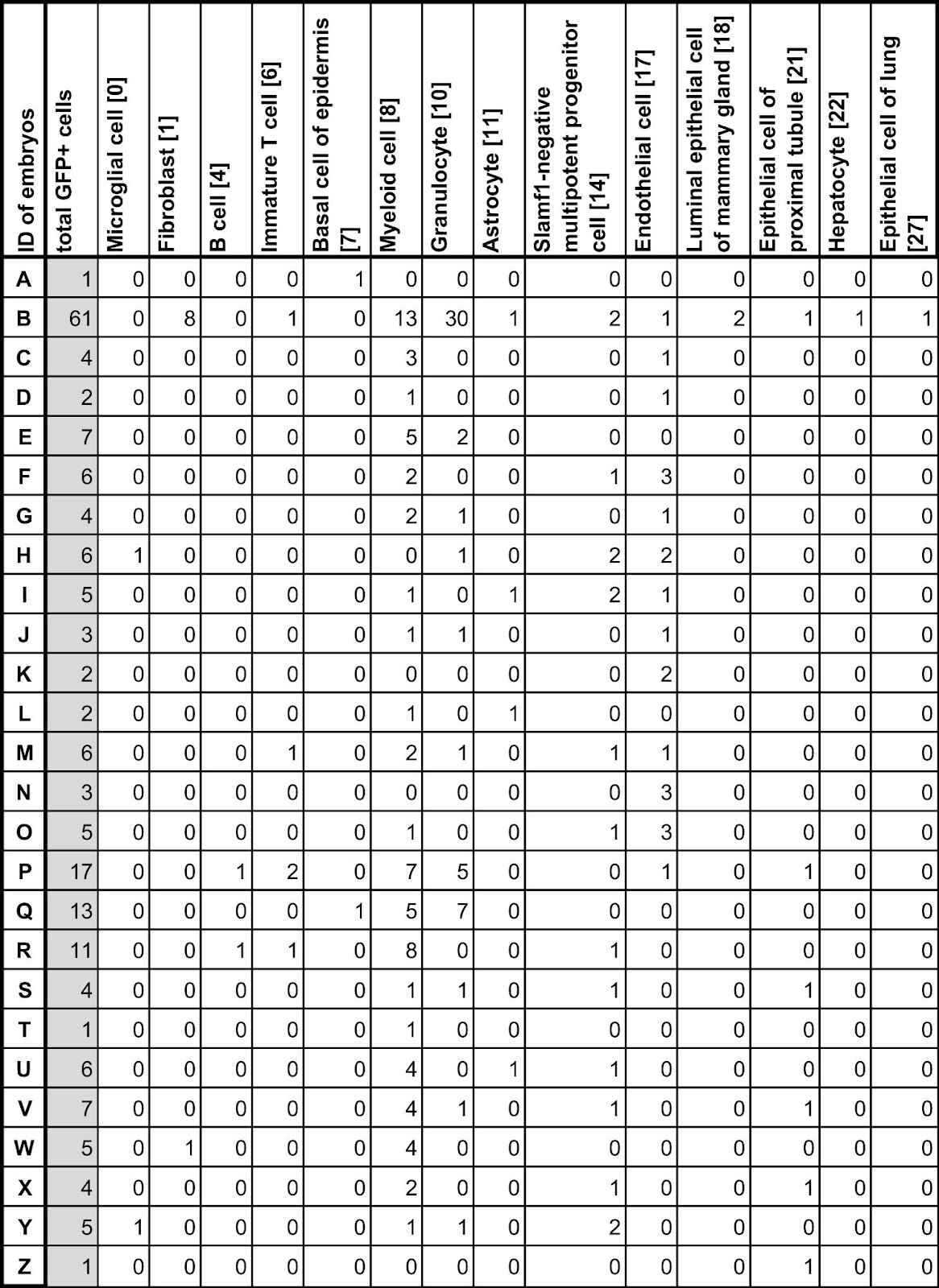

Supplement: Supplementary file 2 — Supplementary Tables. [file 41598_2022_20781_MOESM2_ESM.docx]
